# Transcutaneous auricular vagus nerve stimulation for the treatment of myoarthropatic symptoms in patients with craniomandibular dysfunction – a protocol for a randomized and controlled pilot trial

**DOI:** 10.1186/s40814-024-01447-x

**Published:** 2024-02-08

**Authors:** Lea S. Prott, Frank A. Spitznagel, Alfons Hugger, Robert Langner, Petra C. Gierthmühlen, Mortimer Gierthmühlen

**Affiliations:** 1 Department of Prosthodontics, Medical Faculty and University Hospital Düsseldorf, Heinrich-Heine-University Düsseldorf, Moorenstraße 5, Düsseldorf, 40225 Germany; 2 Institute of Systems Neuroscience, Medical Faculty and University Hospital Düsseldorf, Heinrich Heine University Düsseldorf, Moorenstraße 5, Düsseldorf, 40225 Germany; 3 Institute of Neuroscience and Medicine (INM-7: Brain and Behaviour), Research Centre Jülich, Jülich, 52425 Germany; 4 Department of Neurosurgery, University Medical Center Knappschaftskrankenhaus Bochum, In Der Schornau 23-25, Bochum, 44892 Germany

**Keywords:** Temporomandibular disorders, Myofascial pain, Vagus nerve stimulation, Transcutaneous, Neuromodulation

## Abstract

**Background:**

Temporomandibular disorders (TMD) are a collective term for pain and dysfunction of the masticatory muscles and the temporomandibular joints. The most common types of TMD are pain-related, which may impact the psychological behavior and quality of life. Currently, the most popular methods for the treatment of TMD patients are occlusal splint therapy, often in combination with physical- and/or pharmacotherapy. However, due to the complexity of etiology, the treatment of chronic TMD remains a challenge. Recently, CE-certified systems for non-invasive VNS (transcutaneous auricular vagus nerve stimulation, taVNS) have become available and show positive effects in the treatment of chronic pain conditions, like migraine or fibromyalgia, with which TMD shares similarities. Therefore, it is the main purpose of the study to evaluate the feasibility of daily taVNS against chronic TMD and to assess whether there is an improvement in pain severity, quality of life, and kinetic parameters.

**Methods:**

This study is designed as a single-blinded, double-arm randomized controlled trial (RCT) in a 1:1 allocation ratio. Twenty adult patients with chronical TMD symptoms will be enrolled and randomized to stimulation or sham group. In the stimulation group, taVNS is performed on the left tragus (25 Hz, pulse width 250 µs, 28 s on/32 s off, 4 h/day). The sham group will receive no stimulation via a non-functional identical-looking electrode. Validated questionnaire data and clinical parameters will be collected at the beginning of the study and after 4 and 8 weeks. The compliance of a daily taVNS of patients with chronical TMD will be evaluated via a smartphone app recording daily stimulation time and average intensity. Additionally, the treatment impact on pain severity and quality of life will be assessed with different questionnaires, and the effect on the mandibular mobility and muscle activity will be analyzed.

**Discussion:**

This is the first clinical trial to assess the feasibility of taVNS in patients with chronic TMD symptoms. If taVNS improves the symptoms of TMD, it will be a significant gain in quality of life for these chronic pain patients. The results of this pilot study will help to determine the feasibility of a large-scale RCT.

**Trial registration:**

This study has been registered in the DRKS database (DRKS00029724).

## Introduction

### Background

Temporomandibular disorders (TMD) refer to all neuromuscular and musculoskeletal conditions of the masticatory muscles, temporomandibular joint (TMJ), and the adjacent structures [1], and are mainly characterized by myofascial pain, masticatory muscle pain, and limitations of jaw functions [2]. However, other comorbid types of musculoskeletal pain such as headaches and neck or low back pains frequently occur [3]. The etiology of TMD is multifactorial triggered by biomechanical, neuromuscular, and psychosocial influences [2]. A much higher incidence is reported in women and most prevalent at the age of 30–40 [4]. The role of occlusion in relation to the etiology of TMD is widely considered limited since bruxism appears to be a predisposing and perpetuating factor associated with TMD [5, 6]. Previous studies reported a significant correlation between the occurrence of TMD symptoms and oral parafunctions [7, 8]. The prevalence of treatment need for TMD was estimated to be approximately 15% in the adult population [9], whereas bruxism is even more common with a prevalence of 8–31% [10]. Due to the COVID-19 pandemic, bruxism and orofacial symptoms have increased significantly over the past 3 years [11]. Over 70% of the dentists surveyed in the USA reported a significant increase in the prevalence of bruxism since the onset of the COVID-19 pandemic. Furthermore, the majority of surveyed dentists observed more TMD symptoms as well as chipped and cracked teeth compared to before the pandemic [12]. According to a scientific report released by the World Health Organization (WHO), also the prevalence of anxiety and depression increased by 25% during the first year of the COVID-19 pandemic [13]. Unprecedented stress caused by the lockdown measures and social isolation have been cited as main reasons for this increase. Loneliness, fear of infection, and death as well as financial worries were mentioned as further stressors. Severe mental health problems were most frequently found among younger women (20–45 years) and people with no work and low income [6, 14]. Studies from Israel, Brazil, and Italy [15–17] have shown an increase or worsening of existing TMD symptoms during the pandemic, demonstrating that the relationship between stress exposure and TMD is global [18].

The use of an occlusal splint therapy can be considered the most popular method for the treatment of TMD patients in order to achieve a neuromuscular deregulation and relaxation of the masticatory system [19, 20]. Physical therapy seems to be also effective, especially if the TMD is associated with headache symptoms [21, 22]. The initial management of TMD may include further therapeutic options, such as manual therapy, acupuncture, progressive muscle relaxation, biofeedback, behavioral therapy, or pharmacotherapy (nonsteroidal anti-inflammatory drugs (NSAIDs), tricyclic antidepressants) [21, 23]. However, although the effectiveness of splint therapy has been proven [24], it has limited success when the muscle pain becomes chronic [25]. Moreover, NSAIDs are less effective for chronic masticatory muscle pain [26, 27]. Since pharmacotherapy is often associated with side effects and usually reduces patients’ burden only slightly, new therapeutic methods for the treatment of chronic TMD conditions need to be evaluated.

**Fig. 1:**
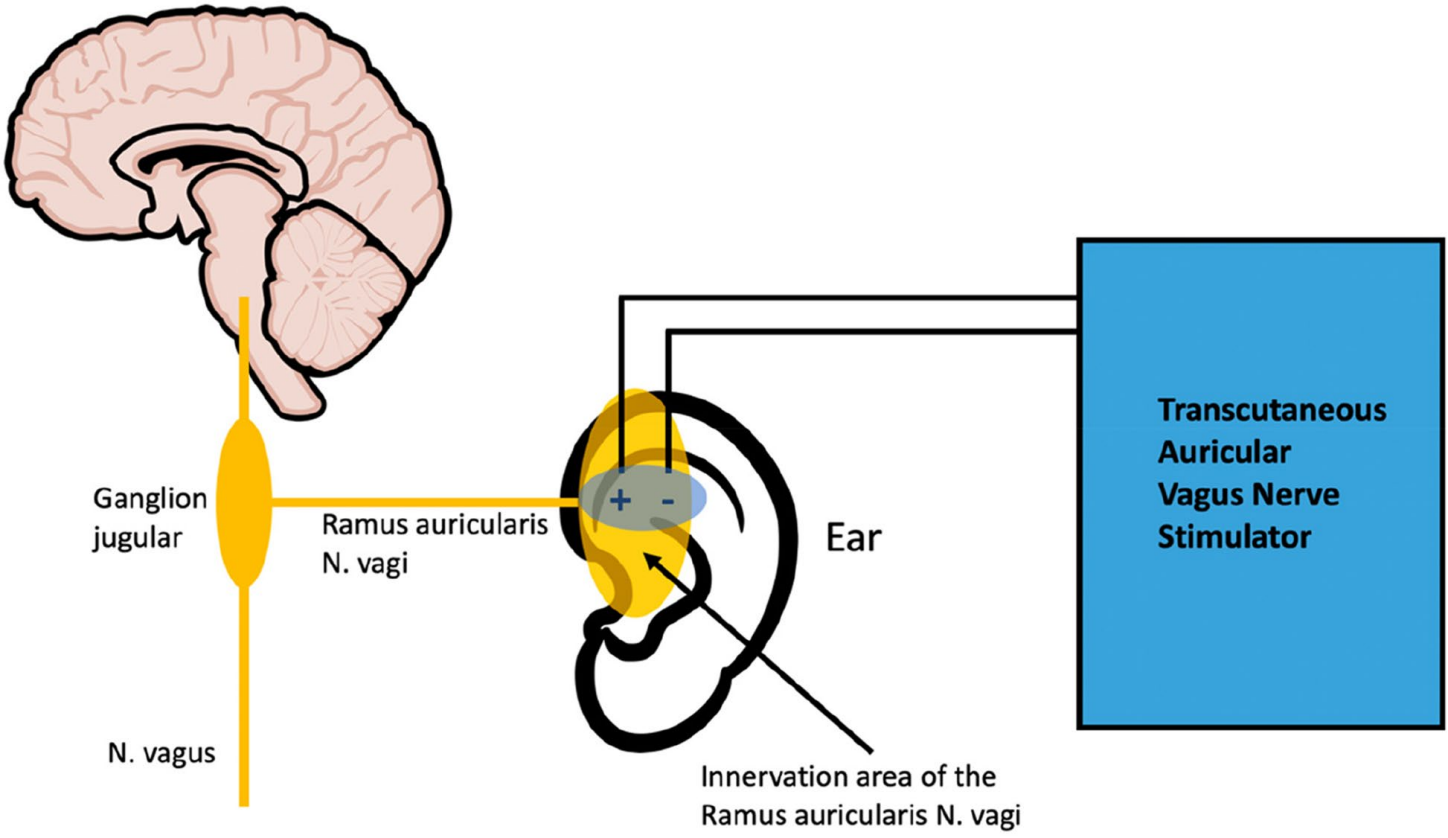
Schematic representation of the effect mechanism of taVNS

Vagus nerve stimulation (VNS) was established three decades ago as an implanted device for the treatment of epilepsy [28]. Nowadays, it has gained further interest since it was approved by the Food and Drug Administration (FDA) for the treatment of major depression in 2005 and cluster headaches in 2017 [29, 30]. The electrical stimulation of vagus nerve afferents causes an activation of the nucleus tractus solitarii as primary central relay (Fig. 1). This nucleus projects via multiple neuronal connections to anatomically distributed subcortical and cortical regions leading to an activation of multiple brain areas which are involved in several inflammatory, nociceptive, and emotional processes [31, 32]. However, although vagus nerve stimulation (VNS) is a well-tolerated treatment option [33], due to its invasive nature, potentially simpler, safer, and less expensive therapies are of interest [34]. The most recent development in the field of vagal neurostimulation are transcutaneous devices allowing external stimulation of the vagus nerve with no need for surgical implantation [35]. This transcutaneous auricular vagus nerve stimulation (taVNS) is based on the assumption that some anatomical structures of the ear, like the tragus or the concha, are innervated by branches of the vagus nerve [36]. By using an auricular electrode, the auricular branch of the vagus nerve is stimulated evoking similar therapeutic effects like the invasive VNS [33]. Studies in patients with chronic migraine and cluster headache, conditions in which trigeminal nociceptive afferents also appear to play a role, showed that taVNS had a positive effect on the headache frequency and severity [37, 38]. A recent review summarized the positive effects of taVNS on acute and chronic pain [39]. Similar to the invasive VNS, taVNS also appears to have an antidepressant effect [40, 41], which may be explained by its influence on the autonomic nervous system in terms of activation of the parasympathetic [42]. Patients with TMD often show depressive symptoms and anxiety, characterized as a form of autonomic dysfunction, which in turn is associated with excessive sympathetic activation and concomitant reduced parasympathetic activation [43]. Due to similarities in the pathophysiology of TMD, taVNS with its positive effect on pain perception and the autonomic nervous system may also have a positive impact on symptoms of a chronical TMD disease and may improve the quality of life of these patients.

### Objectives

#### Primary objectives

Based on the abovementioned observations that taVNS might improve symptoms in both chronic and acute pain, this trial aims to assess the feasibility and compliance of taVNS for patients with TMD. The results will influence the design and methodology of a subsequent large-scale RCT. Specifically, primary study objectives include the following:

1. To collect data to assess the feasibility of the intervention
2. To investigate the compliance of patients with chronic TMD symptoms treated with taVNS
3. To evaluate whether this intervention provides clinically relevant treatment effects regarding pain reduction (GCPS score)

#### Secondary objectives

The secondary objectives of this feasibility trial are to detect potential differences in the outcome variables between stimulation and sham group regarding:

- PHQ-9, GAD-7, PHQ-15, and OHIP-G14 scores
- Clinical signs of TMD (DC/TMD)
- The mandibular range-of-motion capacity
- The electromyographic activity of the main chewing muscles (EMG of masseter muscle and temporalis anterior muscle)

## Methods

**Fig. 2:**
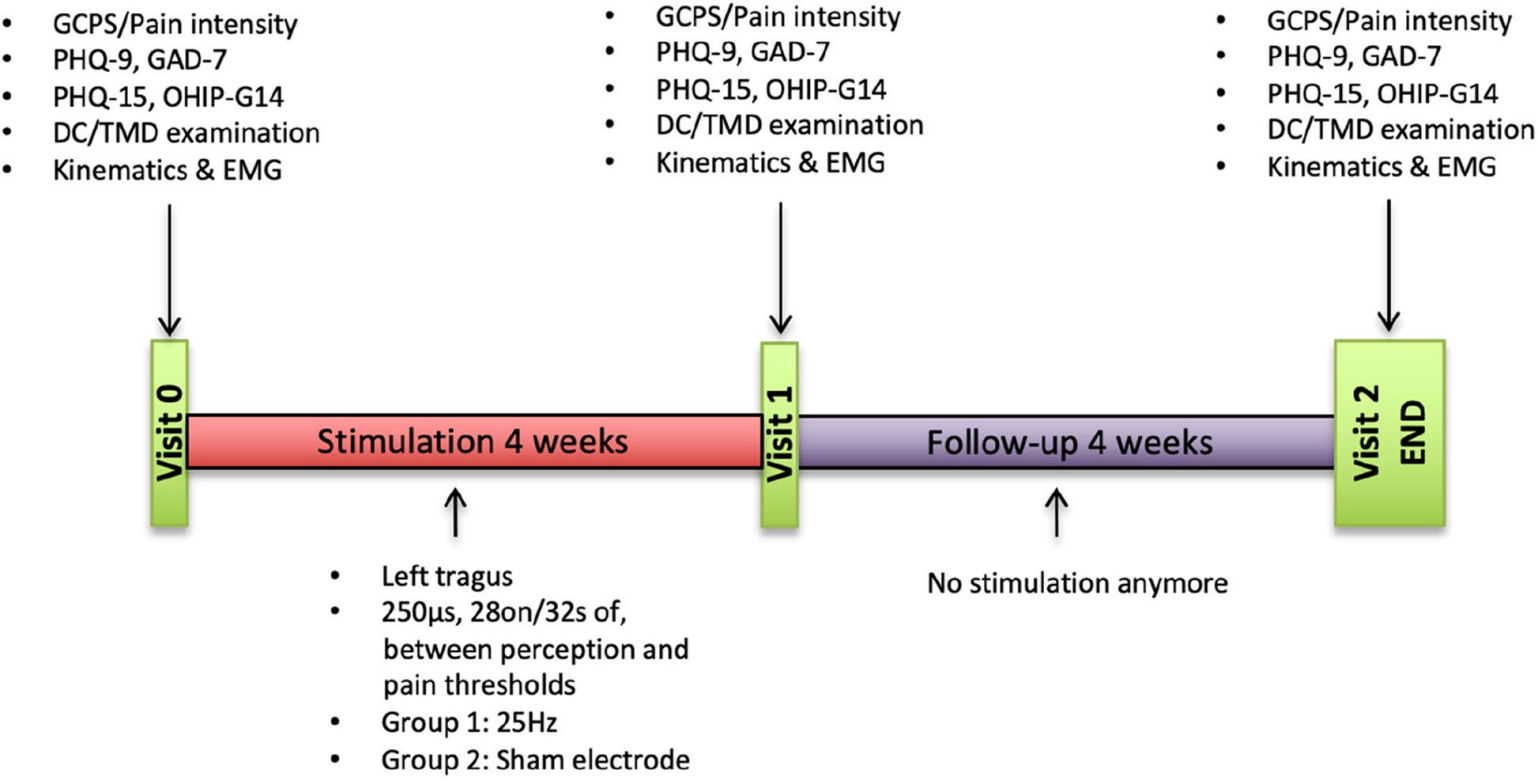
Study periods

The study will be performed at the University Medical Center Düsseldorf (Department of Prosthodontics) in cooperation with the University Medical Center Knappschaftskrankenhaus Bochum (Department of Neurosurgery). A total of 20 patients will be recruited and examined in three study visits (enrolment, visit 0, and 1) during an observation period of 8 weeks (Fig. 2). Study parameters will be determined at 4-week intervals in relation to the baseline measurement. The attached SPIRIT table (Table 1) shows the study period and explains when interventions and questionnaires will be performed within the study.

**Table 1:** Schedule of enrolment, interventions, and assessments

	Study Period			
	Enrolment	Allocation	Post-Allocation	Close-out
TimePoint	-t1	Visit 0	Visit 1	Visit 2
Enrolment				
Eligibility screen	x			
Informed consent	x			
Questionnaires		x	x	x
DC/TMD examination	x	x	x	x
Kinematics		x	x	x
EMG (Electromyography)		x	x	x
Allocation		x		
Installation of digital protocol app on smartphone		x		
Interventions:				
Stimulation				
Sham-Stimulation				
Assessments:				
Download stimulation diary from smartphone app			x	
Baseline questionnaire		x		
Baseline DC/TMD examination	x	x		
Baseline EMG		x		
Baseline Kinematics		x		
Outcome questionnaires			x	x
Outcome DC/TMD examination			x	x
Outcome EMG			x	x
Outcome kinematics			x	x

### Patients

Patients (≥ 18 years) currently treated for chronic TMD conditions (Grade III or IV of the Graduation of Chronic Pain Scale (GCPS) according to von Korff [44]) at the Department of Prosthodontics of the University Medical Center Düsseldorf will be asked to join the study. A certified dentist with experience in TMD diagnosis will perform the examinations according to the diagnostic criteria for TMDs (DC/TMD) [45, 46]. This study is designed as a single-blinded, double-arm randomized controlled trial (RCT) in a 1:1 allocation ratio as a sham and stimulation group will be compared. A concealed randomization and single blinding will be carried out.

Patients are assigned to one of the two parallel treatment groups using a randomization list, which is based on a random, computer-generated numerical sequence. At the first visit, participants will be assigned to their respective group by a researcher who is not involved in patient recruitment.

#### Inclusion criteria

- Chronic temporomandibular disorders (TMD)
- Age ≥ 18 years
- Provided written informed consent to participate in the trial
- Positive response to the question, “Do you have pain in the right side of your face, the left side, or both?” [45]
- Grade III or IV of the Graduation of Chronic Pain Scale (GCPS) [44]
- No or stable depression for at least 4 weeks

#### Exclusion criteria

- Orofacial pain or diagnosis(es) that do not qualify as myalgia, myofascial pain, or arthralgia based on the Diagnostic Criteria for Temporomandibular Disorders (DC/TMD)
- Severe psychiatric disease (e.g., schizophrenia)
- Interventions with vagus nerve stimulation or history of vagotomy
- History of relevant cardiac diseases: bradycardic arrhythmia (e.g., sick sinus syndrome), heart failure, condition after myocardial infarction
- Active implant, e.g., pacemaker, defibrillator, neurostimulator, cochlear implant or drug delivery device, and ventricular shunt
- Inability to understand the study protocol
- Progressive neurological disease (e.g., Parkinson’s disease, MS, epilepsy)
- Pregnancy
- Prostate carcinoma
- Presence of a skin condition like infection, psoriasis, or eczema at the stimulation site
- Presence of any anatomical abnormality preventing successful insertion of the ear electrode
- Presence of any serious medical condition preventing successful study participation
- Acute tinnitus

#### Abort criteria

- Occurrence of an exclusion criteria
- Occurrence of severe cardiac arrhythmias
- Withdrawal of consent

### Data logging

In order to monitor the patient’s compliance, a smartphone app will be installed with the patient’s consent (available for Android and iOS and connected via Bluetooth to the stimulator) for recording of the daily stimulation time and average intensity. A cloud connection or a registration is not necessary. The app should help the patient to keep track of the stimulation times as stimulation should take place 4 h throughout the day. Although the app shows when the recommended stimulation time of 4 h/day has been reached, the stimulator will not automatically seize stimulation after 4 h. After a stimulation period of 4 weeks, the stimulation protocol will be exported and analyzed with respect to mean daily stimulation time and intensity. If the patient does not have a smartphone, it will be provided for the duration of the study.

### Data protection

All data will be recorded pseudonymously and the link between number and patient name will be stored on a different clinical network storage. The electronically recorded data of this study will be maintained on a password-protected database under the control of the principal investigator. All questionnaires and consent forms recorded on paper will be stored in a locked cabinet in a locked room requiring key-card access. All additional information will be stored without any identification of group assignment on a separate database.

### Outcomes

#### Primary outcomes

The primary outcomes will be feasibility, compliance, and the determination of clinically relevant taVNS treatment effects. Feasibility will include recruitment and retention rates, randomization success, blind-success, compliance with questionnaires and assessment procedures and the occurrence of any adverse events (Table 2). The compliance of 4 h/day taVNS will be electronically logged by an app on the patient’s smartphone. Treatment is considered to be compliant when at least 80% of patients use the stimulator for at least 2 h (mean) per day. Furthermore, this study was designed to detect clinically relevant treatment effects, which we consider to correspond to statistically large effects according to Cohen [47]. An established and widely used, validated questionnaire will be applied to assess the pain severity and activity limitations (GCPS (Graduation of Chronic Pain according to Von Korff [44])).

**Table 2:** Key feasibility indicators for progression [48, 49]

Key indicator	Go—proceed with RCT	Amend—proceed with changes	Stop—do not proceed unless changes are possible
**Recruitment rate** *Target figure: 20 participants*	≥ 70% of target number	51–69% of target number	≤ 50% of target number
**Retention rate** *75% of the participants randomized to intervention group will complete outcome measures T0, T1, and T2*	≥ 70% of target number	51–69% of target number	≤ 50% of target number
**Compliance**	≥ 80% of target number use the stimulator for at least 2 h (mean) per day	61–79% of target number use the stimulator for at least 2 h (mean) per day	≤ 60% of target number use the stimulator for at least 2 h (mean) per day

According to power calculations done with G*Power 3.1.9.7 and assuming an alpha error level of 5%, a power of 95%, and a moderate correlation among repeated measures (*r* ≥ 0.5), a sample size of *n* = 18 is required to detect significant intervention (stimulation vs. sham) × time (visit 0, visit 1, visit 2) interaction effects of this size. Assuming a drop-out rate of approximately 10%, a sample of *n* = 20 participants will be initially recruited, 10 in each of the two groups “sham” and “stimulation.” Another just-conducted pilot trial on a taVNS intervention also reaches clinically relevant effects with a similarly small sample size [50]. Furthermore, an investigation of taVNS application in fatigue syndrome reported highly significant effects (*p* < 0.0003) with a sample size of *n* = 15 [51]. Other studies evaluating taVNS application in migraine also used samples of 20 to 30 patients [52–54]. Consequently, a sample size of *n* = 20 appears to be sufficient to achieve statistical significance for clinically relevant (i.e., large) effects of taVNS and was therefore adopted in this study.

#### Secondary outcomes

Secondary objectives focus on potential differences in the outcome variables between the stimulation and sham group regarding changes in the scale of depression (PHQ-9 (Patient Health Questionnaire according to Kroenke et al. [55]), generalized anxiety disorders (GAD-7 (Generalized Anxiety Disorders according to Spitzer et al. [56])), severity of somatic symptoms (PHQ-15 (according to Kroenke et al. [57]), and the oral health-related quality of life (OHIP-G14 (Oral Health Impact Profile G14 according to John et al. [58])). Furthermore, changes in clinical parameters (effect of taVNS on the mandibular range-of-motion capacity (Jaw-Motion-Analyzer, Zebris Medical, Isny/Allgäu, Germany), the electromyographic activity of the main chewing muscles (EMG/WinJaw measuring system (Zebris Medical) of masseter muscle, temporalis anterior muscle) [59–61] and the clinical signs of TMD (DC/TMD examination [46]) will be measured. All examinations will be performed at baseline, after 4 weeks of taVNS treatment and after a further 4 weeks without treatment. It will be assessed, if both subjective and objective impairments can be improved and whether the observed effects last 4 weeks without stimulation.

### Treatment groups

#### Stimulation group

The tVNS-L system is manufactured by tVNS GmbH, Erlangen, Germany, and is CE-certified for left-sided transcutaneous auricular vagus nerve stimulation. Both frequency of 25 Hz and pulse width of 250 µs are fixed and applied within a 28-s on/32-s off protocol. The stimulation intensity can be adjusted by the patient. The system is available on the internet and can be bought in the manufacturer’s online shop without prescription. The stimulation will be performed at the tragus of the left ear in accordance with the CE certificate, and the stimulation intensity is adjusted by the patient just above the perception threshold. Stimulation should be performed throughout the day for 4 h.

#### Sham group

In the sham group, a non-connected fake electrode (sham) with an identical appearance as the real electrode is used for stimulation. At the first visit, the stimulation system is demonstrated to the patient with a functional electrode, and the stimulation is adjusted right above the threshold. Then, the intensity is reduced below the threshold, and the patient is instructed to keep the intensity on that level without altering it during the study. However, the patient will then receive a non-functional electrode which ensures that no stimulation is applied to the ear. Hence, the patient will be blinded, while the dentist knows the allocation.

### Study plan

Patients with TMD conditions are routinely screened with different questionnaires and examination procedures at the Department of Prosthodontics of the University Medical Center Düsseldorf. If a chronical TMD is detected (Grade III or IV of the Graduation of Chronic Pain Scale (GCPS) according to von Korff [44]), the patient could be eligible to participate (inclusion/exclusion criteria), is informed about the study, and receives a study information flyer. If the patient is willing to participate, he/she gets an appointment for the initial visit.

#### Visit 0

At the initial visit, the patient will firstly sign the informed consent and is allocated to a group. Then the questionnaires (GCPS according to Von Korff, PHQ-9, GAD-7, PHQ-15, OHIP-G14) will be filled out by the patient, and the examination according to DC/TMD will be performed. Measurements of the mandibular mobility (range-of-motion) and muscle activity will be performed. Afterwards, the patient will receive the stimulator tVNS-L® and receives a brief training and introduction, then the app is installed on the patient’s smartphone. Finally, he/she is advised to contact the principal investigator if any side effects occur. The stimulation phase for 4 weeks starts. After 1 week, the patient will be contacted by one of the examiners to ensure that no further questions or problems occur during the first days of application.

#### Four-week stimulation phase

##### Visit 1 (after 4 weeks)

At the first visit, the patient will fill out the questionnaires again (GCPS according to Von Korff, PHQ-9, GAD-7, PHQ-15, OHIP-G14). The DC/TMD examination and the acquisition of the mandibular mobility (range-of-motion) and muscle activity by the appropriate measurement systems will be performed again. Under the patient’s supervision, the stimulation statistics are exported from the smartphone app, and the stimulator is given back to the examiner.

##### Visit 2 (after another 4 weeks)

The last visit will be another 4 weeks later. In this follow-up phase, it should be investigated whether the effect of taVNS is sustained or rapidly washed out in order to evaluate the longer-term therapy success. At the last visit, patients will answer the questionnaires (GCPS according to Von Korff, PHQ-9, GAD-7, PHQ-15, OHIP-G14) for the third time, and the DC/TMD examination, the measurements of mandibular mobility (range-of-motion) and muscle activity will also be performed again.

### End of study

#### Data analysis

This trial features a 2 × 3 factorial design with the factors group (stimulation, sham) and time (Visits 0, 1, 2). Main and interaction effects on the secondary endpoints will be statistically tested via a mixed-measures ANOVA. In case the preconditions for ANOVA use are not met, nonparametric alternatives will be employed. Global effects will be further elucidated by appropriate post hoc comparisons between individual conditions (i.e., per group or time point). The significance threshold will be set to *p* < 0.05, corrected for multiple comparisons where needed. Furthermore, estimates of treatment effect size and confidence intervals will be reported in addition to significance testing results. These analyses will provide a treatment effect estimate on each outcome measure. Outcome measures at baseline, 4 and 8 weeks post-intervention will be entered into the model as the dependent variables with fixed effects of study arm, baseline outcome measures, time, and time point by study arm interaction.

## Discussion

The strength of this protocol is the novelty of investigating taVNS as a potential, well-tolerated, and safe treatment option [33] for patients with chronic TMD symptoms. Further advantages of taVNS are that it can be combined with other treatment options, like splint-, physical- or pharmacotherapy, without risking adverse side effects. Moreover, due to its ear pod-style design, patients may continue their routine activities, facilitating a high treatment compliance [38].

Several studies are available in which healthy participants were subjected to an acute taVNS. Importantly, none of the studies reported an occurrence of relevant side effects in the short or longer run [34, 39, 62, 63]. Only nausea, skin irritation of the ear, and worsening of preexisting tinnitus were observed. The drop-out rate associated with side effects was only 2.6% [64–66].

Due to the complexity of the etiology, the diagnosis and treatment of TMD remain a challenge [21]. A high vagal activity has been considered to improve the psychological health and is hypothesized to have therapeutic potential in a wider spectrum of illnesses [34, 43]. Since vagus nerve stimulation has already been investigated successfully for several other acute and chronic pain disorders with similarities in the pathophysiology of TMD, including migraine, depression, tinnitus, and fibromyalgia [39], a response to the innovative and gentle therapy option of taVNS is possible. Potentially, taVNS could also give chronic TMD pain patients a significant gain in their quality of life, which makes its non-invasive application justifiable.

Potential limitations of the present study include that the taVNS requires the active patient cooperation compared to an implantable VNS [35]. However, due to the practical design of taVNS and the high level of suffering of TMD patients, the patients’ compliance should be high. A further limitation is the single-blinded study design. A future definitive trial should be double-blinded with an independent dentist performing the device instructions and blinded outcome assessors. Furthermore, results will only reflect short- or mid-term effects as the treatment duration will be only 4 weeks [40]. Future studies should also investigate long-term effects.

## Data Availability

As this is a report of our trial protocol, no data is available yet.

## Abbreviations

DC/TMD: Diagnostic criteria for temporomandibular disorders
EMG: Electromyography
FDA: Food and Drug Administration
GAD: Generalized Anxiety Disorders
GCPS: Graduation of Chronic Pain
iVNS: Invasive vagus nerve stimulation
NSAIDs: Nonsteroidal anti-inflammatory drugs
OHIP: Oral Health Impact Profile
PHQ: Patient Health Questionnaire
RCT: Randomized controlled trial
TMD: Temporomandibular disorders
TMJ: Temporomandibular joint
taVNS: Transcutaneous auricular vagus nerve stimulation
VNS: Vagus nerve stimulation
WHO: World Health Organization
